# Trait and Marker Associations in *Oryza nivara* and *O. rufipogon* Derived Rice Lines under Two Different Heat Stress Conditions

**DOI:** 10.3389/fpls.2017.01819

**Published:** 2017-10-26

**Authors:** V. Vishnu Prasanth, M. Suchandranath Babu, Ramana K. Basava, V. G. N. Tripura Venkata, Satendra K. Mangrauthia, S. R. Voleti, Sarla Neelamraju

**Affiliations:** Indian Institute of Rice Research (ICAR), Rajendranagar, Hyderabad, India

**Keywords:** heat tolerance, wild rice, introgression lines (ILs)

## Abstract

Wild species and derived introgression lines (ILs) are a good source of genes for improving complex traits such as heat tolerance. The effect of heat stress on 18 yield traits was studied in four treatments in two seasons, under field conditions by subjecting 37 ILs and recurrent parents Swarna and KMR3, N22 mutants, and wild type and 2 improved rice cultivars to heat stress using polycover house method in wet season and late sowing method in dry season. Normal grown unstressed plants were controls. Both correlation and path coefficient analysis showed that the major contributing traits for high yield per plant (YPP) under heat stress conditions were tiller number, secondary branches in panicle, filled grain number, and percent spikelet fertility. Three ILs, K-377-24, K-16-3, and S-148 which gave the highest YPP of 12.30–32.52 g under heat stress in both the seasons were considered the most heat tolerant. In contrast, K-363-12, S-75, and Vandana which gave the least YPP of 5.36–10.84 g were considered heat susceptible. These lines are a good genetic resource for basic and applied studies on heat tolerance in rice. Genotyping using 49 SSR markers and single marker analysis (SMA) revealed 613 significant marker- trait associations in all four treatments. Significantly, nine markers (RM243, RM517, RM225, RM518, RM525, RM195, RM282, RM489, and RM570) on chromosomes 1, 2, 3, 4, 6, and 8 showed association with six traits (flag leaf spad, flag leaf thickness, vegetative leaf temperature, plant height, panicle number, and tiller number) under heat stress conditions in both wet and dry seasons. Genes such as heat shock protein binding *DnaJ, Hsp70*, and temperature-induced lipocalin-2 *OsTIL-2* close to these markers are candidates for expression studies and evaluation for use in marker assisted selection for heat tolerance.

## Introduction

The global mean air temperatures are expected to rise by 2–4.8°C in the next few decades as a result of global warming (IPCC, 2013). Rice is an important cereal crop which provides food security for more than 60 per cent of the world population. Most of the rice growing areas of tropical and subtropical regions have temperature close to the threshold temperature of 33°C required for rice (Teixeira et al., 2013). High temperatures both during day and at night cause considerable loss in rice yield (Peng et al., 2004; Jagadish et al., 2010a,b; Kadam et al., 2014). Temperature above 35°C for more than 60 min during anthesis results in high sterility among panicles in rice (Jagadish et al., 2007). High temperature stress during flowering causes abnormal anther dehiscence (Matsui and Omasa, 2002), poor pollen germination (Jagadish et al., 2010b), and reduced grain filling duration (Cao et al., 2016). All these lead to increased spikelet sterility and yield loss. Varieties differ in their response to high temperature. Moroberekan, a heat-sensitive genotype showed 82% spikelet sterility but Nagina22 (N22), a highly tolerant genotype showed only 29% spikelet sterility at 38°C (Jagadish et al., 2010b). The extent of reduction in rice yield due to heat also depends on several other factors such as the duration and intensity of heat stress, the growth stage at which heat episode occurs, vapor pressure deficit, and the water status of plants.

Heat tolerance is a complex trait which involves various morphological, physiological, biochemical, and molecular changes in plants. There are several reports on the effects of high temperature and response of rice (Sailaja et al., 2015; Shi et al., 2015; Ye et al., 2015a,b; Brito et al., 2016; Cao et al., 2016; Tanamachi et al., 2016; Wu et al., 2016; Zhang et al., 2016). Majority of these studies considered spikelet fertility (SF) as major criterion for assessment of heat tolerance. Several quantitative trait loci (QTLs) have also been mapped for heat tolerance at booting, flowering and grain filling stages in rice (Ishimaru et al., 2016). In general, QTLs were mapped for SF under heat stress. Among all the QTLs, only one QTL (*qHTSF4.1*) has been validated in different genetic backgrounds and also fine mapped (Ye et al., 2015a,b). In our previous study, using association mapping analysis we validated nine markers for five traits [SF, yield per plant (YPP), Heat Stability Index (HSI) of SF and HSI of YPP] under heat stress (Prasanth et al., 2016). Evidence was provided that SF alone is not the best criterion to assess heat tolerance in rice and yield should also be included. Rice lines with low SF but high YPP and vice versa were identified under two different heat stress conditions (Prasanth et al., 2016). Hence, a thorough understanding on the contribution of various morphological and yield related traits toward heat tolerance and association of the markers with those traits is required for efficient selection and development of heat tolerant rice lines.

Most of the studies on heat tolerance in rice are based on exposing the plants to high temperature in green houses or temperature controlled growth chambers. There are very few studies on effects of heat stress on rice under near—natural field conditions. In field, the temperature varies dynamically as do the other environmental factors such as CO_2_ concentration, relative humidity, vapor pressure deficit (VPD), light intensity, wind velocity, and so the response to temperature is often confounded (Poorter et al., 2016). Reduction in grain yield due to heat stress is comparatively less in controlled chambers than in natural field conditions (Hall, 2011). Also, the impact of long duration stress upon the plants is quite different than short duration stress. In long duration stress, plants can acclimatize to high temperature stress beginning from vegetative stage itself. To include the variability in acclimation exhibited by plants, an in depth field based screening is needed.

The wild progenitor species *Oryza nivara* and *Oryza rufipogon* which cross easily with cultivated rice comprise an easily exploitable and diverse gene pool for rice improvement. Introgression lines (ILs) developed by crossing elite rice lines with these two wild species showed lines with high yield and tolerance to salinity and drought (Swamy et al., 2014; Sreenu et al., 2015; Pushpalatha et al., 2016; Haritha et al., 2017). Screening of these lines for heat tolerance would help in the identification or development of new heat tolerant genotypes. The objective of the present study was to (1) screen elite × wild ILs for heat tolerance under field conditions based on morphological and yield related traits, (2) analyse direct and indirect effect of these traits on grain yield and, (3) identify markers and candidate genes linked to traits under heat stress.

## Materials and methods

A set of 48 stable lines consisting of 16 Swarna × *O. nivara* introgression lines (Swarna ILs), 18 KMR3 × *O. rufipogon* introgression lines (KMR3 ILs), 4 EMS induced mutants of Nagina 22 (N22) (NH219, NH363, NH686, NH787) and seven wild rice/landraces/improved varieties viz., *O. rufipogon* (WR120), *O. nivara* (IRGC 81848), IR64, Vandana, BPT5204, Azucena and Nipponbare were used in the present study. Swarna is a popular mega rice cultivar, KMR3 is a restorer line of the popular 3-line rice hybrid KRH2 and N22 is a well-known heat tolerant aus variety. The details of all the lines are given in Supplementary Table 1.

### Phenotyping

The experiments were conducted at IIRR field (latitude and longitude: 17° 22′31″ N and 78° 28′27″E) during wet season 2012 (July to December) using poly cover house method and dry season 2013 (January to May) using late sown method (flowering coinciding with high temperature) for heat stress following Prasanth et al. (2016). The respective controls were normal conditions without poly cover in 2012 and sown in normal sowing time in 2013. In wet season 2012, temperature in control conditions was ≤33, ≥24.5°C, and mean was 30°C during day time. The RH in control conditions was ≥30, ≤97%, and mean was 87%. The temperature inside poly cover house was ≥30.2, ≤48°C, and mean was 44.3°C during day time. In dry season, the temperature during day time in normal sowing method was ≤38.9, ≥37.6°C, and mean was 35.7°C while in late sown method it was ≤42.33, ≥40.0°C, with mean of 37.6°C. The RH observed in both normal and late sown methods was not significantly different.

The following 18 morphological and yield traits were measured in both treatments in both seasons.

(1) Vegetative leaf spad value (VLS) (2) Flag leaf spad value (FLS) (3) Vegetative leaf thickness (VLTH) (4) Flag leaf thickness (FLTH) (5) Vegetative leaf temperature (VLT) (6) Flag leaf temperature (FLT) (7) Time for 50% flowering (FT) (8) Plant height (PH) (9) Tiller number per plant (TN) (10) Panicle number per plant (PN) (11) Panicle length (PL) (12) Primary branches per panicle (PB) (13) Secondary branches per panicle (SB) (14) Filled grain number per panicle (FGN) (15) % Spikelet fertility (%SF) (16) Total grain number (TGN) (17) Biomass per plant (BM) (18) grain yield per plant (YPP).

### Genotyping

Genomic DNA was isolated from fresh leaves of all the 48 lines using CTAB method and genotyped using 49 SSR markers which are previously reported to be linked or close to QTLs for spikelet fertility or pollen fertility under heat stress (Prasanth et al., 2016). The details of all these markers are available at http://www.gramene.org/markers/microsat.

### Statistical analysis

Descriptive statistics, Pearson correlation analysis among 18 agronomic and yield traits were performed individually for control and heat stress conditions (cover house/late sown) in wet season 2012 and dry season 2013 using Statistix 8.1. Percent increase or decrease in the trait values under high temperature conditions was also calculated. Path coefficient analysis using R programming version 3.2.3 using package agricolae was performed to identify the direct and indirect effects of component traits on grain yield. Single marker analysis (SMA) was performed by one way Anova using MINITAB V14.0 (Minitab Inc., USA) to find out the association of each marker with the traits.

### Candidate gene analysis

Candidate genes were identified in case of markers associated with traits only under heat stress conditions in both wet and dry seasons by obtaining physical position of the markers from Gramene data base (http://www.gramene.org/). The stress response putative candidate genes were identified in the genomic region 1 Mb upstream and 1 Mb downstream of these marker positions using RAPDB (http://rapdb.dna.affrc.go.jp/).

## Results

### Phenotypic performance

Descriptive statistics for 18 morphological and yield traits under wet season normal (WN), wet season heat stress (WH) in 2012, dry season normal (DN), dry season heat stress i.e., late sown (DH) in 2013 are shown in Supplementary Table 2.

All traits followed normal distribution except FT in control during wet season and FLT and %SF in control during dry season. In wet season, the mean values increased significantly under heat stress for VLS (by 7.09%), VLTH (by 30.8%), FLT (by 28.26%) and PH (by 9.55%) but decreased significantly for TN (by 1.34), FGN (by 37.7), %SF (by 23.45), and YPP (by 45.68%). In dry season, the mean values increased significantly under heat stress for FLTH (by10.87%), VLT (by 5.54%), and PL (by 4.04%) but reduced significantly for VLS (by 2.57%), VLTH (by 20.4%), FLT (by 11.66%), and %SF (by 11.05%). Though, YPP reduced under heat stress conditions during both wet (by 45.68%) and dry (by 15%) seasons but reduction was significant only during wet season. In wet season all lines flowered earlier (by ~5 days) under heat stress when compared with flowering in control but in dry season, the average flowering time of all genotypes was more (by ~5 days) under heat stress than in control. The trait mean values under heat stress in case of three traits, VLS, VLTH, and FLT reduced significantly during wet season but increased significantly during dry season.

The effects of season (wet and dry), temperature (control vs. heat stress), genotypes (36 ILs), and their interactions on 18 traits are presented in Table 1. There were significant variations among genotypes and also in interactions of genotype × temperature × season for all traits. All traits except VLTH showed highly significant differences with respect to season. There were significant differences among treatments for all traits except FLS, VLTH, FL, PB, and BM. The effects of genotype × temperature and genotype × season were significant for all traits except FT and YPP. SF, and YPP showed significant differences with all seven sources of variances except genotype × temperature and genotype × season did not show significant differences in YPP.

**Table 1:** Analysis of variance of 18 agronomic and yield traits under normal conditions and polycover house/late sown conditions during wet season 2012 and dry season 2013.

**Source of variation**	**df**	**VLS**	**FLS**	**VLTH (mm)**	**FLTH (mm)**	**VLT (°C)**	**FLT (°C)**	**FT (days)**	**PH (cm)**	**TN**	**PN**	**PL (cm)**	**PB**	**SB**	**FGN**	**%SF**	**TGN**	**BM (g)**	**YPP (g)**
Season	1	355.45^***^	191.46^***^	1.56^NS^	225.15^***^	2339.59^***^	526.5^***^	289.97^***^	3061.45^***^	275.47^***^	250.38^***^	46.02^***^	67.22^***^	10.07^***^	156.71^***^	233.11^***^	29.29^***^	16.81^***^	21.23^***^
Genotypes	35	6.99^***^	9.69^***^	3.21^***^	4.13^***^	8.42^***^	11.26^***^	5.12^***^	139.35^***^	5.96^***^	5.59^***^	17.68^***^	12.76^***^	24.93^***^	17.36^***^	7.25^***^	31.76^***^	15.73^***^	3.51^***^
Temperature	1	10.89^***^	1.71^NS^	1.8^NS^	91.52^***^	1860.79^***^	272.06^***^	0.04^NS^	256.41^***^	14.93^***^	16.16^***^	6.84^**^	0.05^NS^	61.35^***^	224.85^***^	285.71^***^	23.64^***^	2.94^NS^	67.59^***^
Genotypes^*^ Temperature	35	4.65^***^	5.23^***^	3.19^***^	2.1^***^	9.07^***^	6.92^***^	0.94^NS^	10.66^***^	1.63^*^	1.75^**^	3.29^***^	2.19^***^	4.62^***^	5.9^***^	4.44^***^	5.66^***^	3.36^***^	1.37^NS^
Season^*^Genotypes	35	4.79^***^	6.02^***^	3.53^***^	3.63^***^	7.44^***^	7.16^***^	1.03^NS^	48.59^***^	3.75^***^	4.08^***^	5.23^***^	4.17^***^	5.04^***^	6.92^***^	4.01^***^	6.9^***^	8.74^***^	1.08^NS^
Season^*^Temperature	1	73.10^***^	46.05^***^	415.04^***^	0.00^NS^	1062.25^***^	1906.27^***^	22.98^***^	43.98^***^	9.37^**^	6.81^**^	13.08^***^	1.8^NS^	19.17^***^	68^***^	26.5^***^	55.97^***^	115.25^***^	16.8^***^
Season^*^Genotypes^*^Temperature	35	3.34^***^	4.92^***^	3.59^***^	2.29^***^	8.25^***^	5.32^***^	6.32^***^	8.94^***^	2.19^***^	2.13^***^	3.01^***^	1.55^*^	3.18^***^	2.6^***^	3.26^***^	3.76^***^	4.87^***^	3.12^***^

*** Significant at 0.001 level of probability;

** Significant at 0.01 level of probability;

* *Significant at 0.05 level of probability. VLS, Vegetative leaf spad value; FLS, Flag leaf spad value; VLTH, Vegetative leaf thickness; FLTH, Flag leaf thickness; VLT, Vegetative leaf temperature; FLT, Flag leaf temperature; FT, Time for 50% flowering; PH, Plant height; TN, Tiller number per plant; PN, Panicle number per plant; PL, Panicle length; PB, Primary branches per panicle; SB, Secondary branches per panicle; FGN, Filled grain number per panicle; %SF, Percent spikelet fertility; TGN, Total grain number; BM, Biomass per plant; YPP: grain yield per plant*.

Based on absolute values of all 18 traits (Supplementary Table 3), the top 11 genotypes with high YPP (>12.21 g) in WH 2012 flowered early (96–03 days) except Swarna IL S-65 (112 days). These early flowering high yielding genotypes exhibited high values for PH, TN, PN, PL, SB, FGN, %SF, and BM and low values for VLS and FLTH. Out of top 10 genotypes showing high YPP, five genotypes (KMR3, K-40, K-50, K-16-3, and S-65) also showed high BM (>27 g) whereas four genotypes (K-198, K-103, K-377-24, and K-137) had high SF % (>76%). These results indicate the importance of both BM and %SF in contributing to high YPP. However, K-13-5 which showed high values for both %SF (86.76%) and BM (27.06 g) showed less YPP (10.85 g). In DH, the top 11 genotypes, exhibiting high YPP (>20.48 g) flowered late and flowering time ranged from 113 to 144 days. These genotypes showed high VLS, TN, FGN, and %SF and they had low VLT. The SF of these lines ranged from 88 to 91%. Out of all 48 lines, Swarna IL S-248 (released as DRR Dhan 40) showed high values for three important traits, YPP, BM and %SF. KMR3 IL K-13-7 showed high YPP and BM and four ILs (K-377-24, K-458, S-250, and S-148) showed high YPP and %SF.

These results indicate that the higher yielding ILs had in general either higher BM or %SF or both but the reverse is not true. S-24 which had high %SF (88.6%) and BM (26.38 g) showed less YPP (17.02 g). Tiller number and FGN were the common traits that contributed to higher yield in both WH and DH treatments. Three ILs (K-377-24, K-16-3, and S-148) were considered to be heat tolerant as they were in the list of top 11 genotypes with high YPP in both WH and DH conditions. On the contrary K-363-12, S-75, and Vandana were considered as heat susceptible as they were in the list of 11 genotypes with least YPP in both WH and DH conditions. Genotypes with more YPP in WH showed low VLS in WH but genotypes with more YPP in DH showed high VLS in DH. It may be noted that the leaf temperature was low during the vegetative phase in DH and reproductive phase in WH.

### Pearson correlation analysis

**Figure 1:**
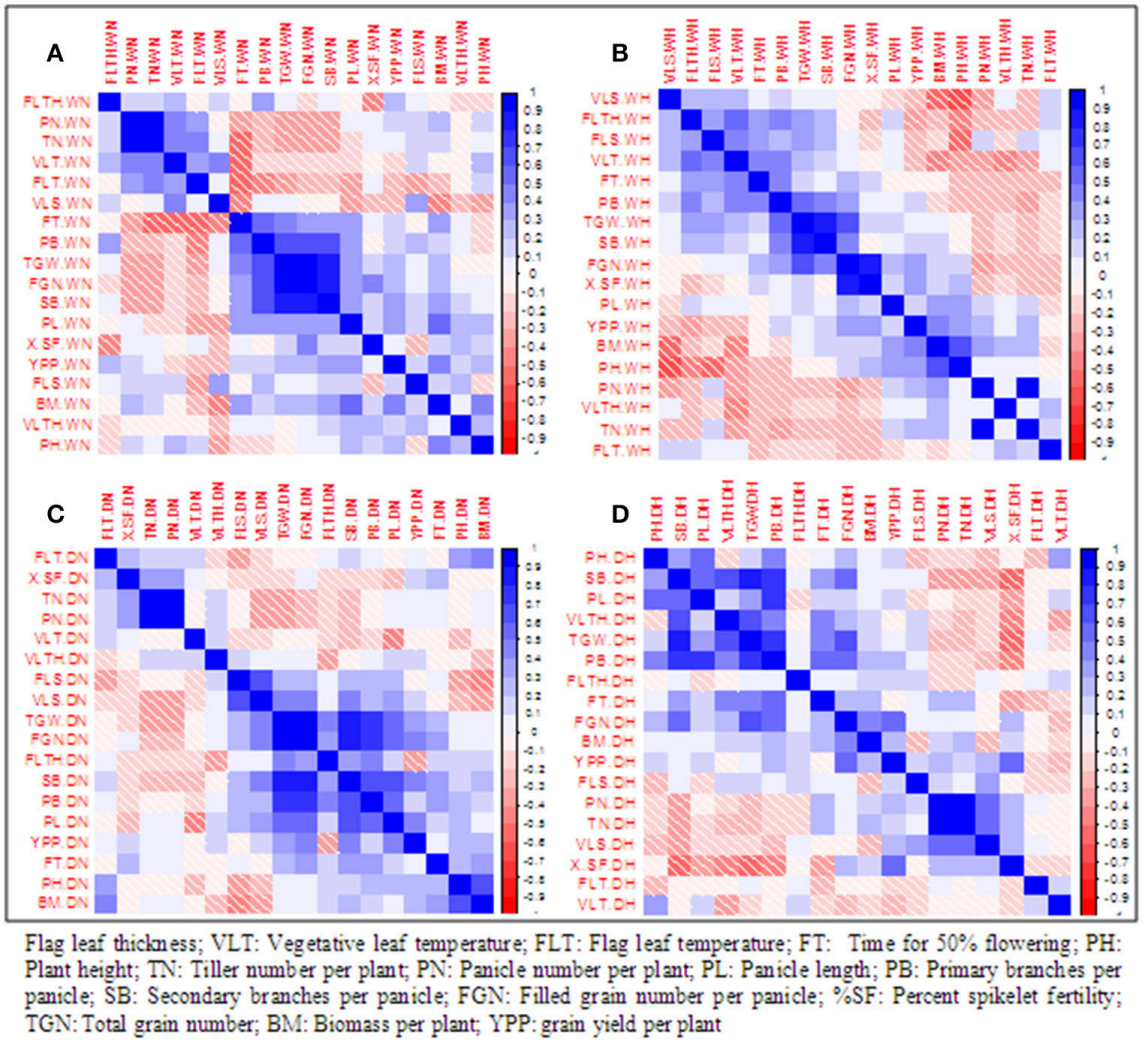
Pearson correlation among 18 traits under normal conditions and polycover house /late sown conditions during wet season 2012 and dry season 2013. **(A)** WN, Wet season normal; **(B)** WH, wet season heat stress; **(C)** DN, Dry season normal; **(D)** DH, Dry season heat stress.

Pearson correlation among 18 traits was computed separately in two treatments and two seasons (Figure 1). During wet season, YPP was correlated positively and significantly with BM, SB, and PL in control but with BM, FGN, and %SF in heat stress. During dry season, YPP was significantly correlated with PL, FGN, and TGN in control but with FGN and %SF in heat stress. Thus, in heat stress, YPP was correlated with both FGN and %SF in both seasons. There was a significant negative correlation between YPP and FLTH in WH but in dry season, this negative correlation was observed only in control. YPP was positively correlated with FGN in all treatments except in WN. It was also significantly correlated with BM in both control and heat stress during wet season but not in any treatment in dry season. It is noteworthy that %SF was significantly correlated with YPP and FGN only under heat stress but not in control during both seasons.

### Path coefficient analysis

As simple correlation does not provide the true contribution of the traits to yield, these correlations were partitioned into direct and indirect effects through path coefficient analysis. The estimates of direct and indirect effects of 17 agronomic and yield related traits on YPP by path coefficient analysis are provided separately as two treatments and two seasons in Table 2. During wet season control conditions, FLTH, TN, SB, and TGN were highly correlated with YPP directly whereas FT, PB, FGN, and TGN contributed to yield indirectly via SB and FGN and SB via TGN. There was significant indirect effect of PL on YPP via TN. PB and FGN were negatively correlated with yield directly. Under wet season heat stress, FLT, PN, SB, and FGN were highly and positively correlated directly to YPP whereas FLS, PH, TN, and %SF were highly negatively correlated with YPP. Even though, TN itself had negative and direct effect on YPP, but 9 out of 17 traits contributed positively indirectly via TN to YPP. These results on TN were in contrast with results on PN. PN had highly positive and direct effect on YPP but eight traits had negative effects on YPP via PN. During dry season, under control conditions, TGN and BM had high positive and direct effects whereas PB and %SF showed high negative and direct effects on YPP. Eight traits showed high positive indirect effects on YPP via TGN. Under late sown conditions, PN, PL, and %SF showed high positive and direct effects on YPP. Even though, TN showed high negative and direct effect on YPP, it showed high positive indirect effect via PN.

**Table 2:** The direct and indirect contribution of 17 agronomic traits to yield per plant (YPP) under normal conditions and polycover house/late sown conditions during wet season 2012 and dry season 2013.

**Trait/Treatment**	**VLS**	**FLS**	**VLTH (mm)**	**FLTH (mm)**	**VLT (°C)**	**FLT (°C)**	**FT (days)**	**PH (cm)**	**TN**	**PN**	**PL (cm)**	**PB**	**SB**	**FGN**	**%SF**	**TGN**	**BM (g)**	**Total effect**
**WET SEASON 2012 NORMAL CONDITIONS**																		
VLS	−**0.033**	0.086	0.023	0.104	−0.147	−0.026	−0.136	−0.149	0.083	0.004	−0.029	−0.028	−0.066	0.038	0.023	−0.017	0.126	−0.260
FLS	−0.010	**0.276**	−0.019	0.023	−0.067	0.130	−0.003	−0.058	0.120	−0.072	0.014	−0.196	0.186	0.000	0.053	−0.020	−0.042	0.169
VLTH (mm)	0.008	0.055	−**0.093**	−0.081	0.017	0.011	0.003	0.111	−0.055	0.004	0.018	−0.063	0.199	−0.048	−0.006	0.019	−0.009	0.178
FLTH (mm)	−0.006	0.011	0.013	**0.575**	−0.073	0.015	0.000	−0.067	0.138	−0.080	0.000	−0.259	0.093	0.095	0.029	−0.031	0.003	0.230
VLT (°C)	−0.015	0.055	0.005	0.127	−**0.333**	−0.133	−0.162	0.111	0.442	−0.181	−0.012	0.070	−0.159	0.114	−0.064	−0.005	0.021	−0.119
FLT (°C)	−0.002	−0.097	0.003	−0.023	−0.120	−**0.371**	−0.184	0.082	0.424	−0.143	−0.032	0.280	−0.371	0.238	−0.182	0.004	0.069	−0.285
FT (days)	0.014	−0.003	−0.001	0.000	0.170	0.215	**0.317**	−0.058	−0.479	0.169	0.033	−0.357	0.517	−0.380	0.258	0.001	−0.108	0.184
PH (cm)	0.010	−0.033	−0.021	−0.081	−0.077	−0.063	−0.038	**0.481**	0.083	−0.051	0.023	0.119	0.013	−0.067	0.000	0.015	−0.135	0.291
TN	−0.003	0.036	0.006	0.086	−0.160	−0.171	−0.165	0.043	**0.922**	−0.409	−0.011	0.196	−0.491	0.333	−0.229	0.004	−0.042	0.068
PN	0.000	0.047	0.001	0.109	−0.143	−0.126	−0.127	0.058	0.894	−**0.422**	0.000	0.154	−0.411	0.304	−0.211	0.004	−0.087	0.159
PL (cm)	0.012	0.047	−0.021	0.000	0.047	0.145	0.127	0.135	−0.120	0.000	**0.083**	−0.252	0.438	−0.333	0.188	0.014	−0.167	0.343^*^
PB	−0.001	0.077	−0.008	0.213	0.033	0.148	0.162	−0.082	−0.258	0.093	0.030	−**0.701**	0.928	−0.571	0.398	−0.001	−0.060	0.182
SB	0.002	0.039	−0.014	0.040	0.040	0.104	0.124	0.005	−0.341	0.131	0.027	−0.490	**1.326**	−0.799	0.522	0.008	−0.105	0.397^*^
FGN	0.001	0.000	−0.005	−0.058	0.040	0.093	0.127	0.034	−0.323	0.135	0.029	−0.420	1.114	−**0.951**	0.568	0.031	−0.075	0.226
%SF	−0.001	0.025	0.001	0.029	0.037	0.115	0.140	0.000	−0.360	0.152	0.027	−0.476	1.180	−0.923	**0.586**	0.015	−0.072	0.258
TGN	0.008	−0.077	−0.024	−0.247	0.023	−0.019	0.003	0.096	0.046	−0.021	0.016	0.007	0.146	−0.409	0.117	**0.073**	−0.030	0.010
BM (g)	0.014	0.039	−0.003	−0.006	0.023	0.085	0.114	0.216	0.129	−0.122	0.046	−0.140	0.464	−0.238	0.141	0.007	−**0.299**	0.453^**^
**WET SEASON 2012 POLYCOVER HOUSE CONDITIONS (HEAT STRESS)**																		
VLS	−**0.404**	−0.123	−0.019	−0.099	−0.070	0.200	0.007	0.665	1.248	−1.401	−0.041	0.009	0.045	0.000	−0.017	0.094	−0.126	−0.213
FLS	−0.085	−**0.585**	0.078	−0.175	−0.138	0.094	0.092	0.588	−0.602	0.525	0.008	0.024	0.440	0.060	−0.077	0.222	−0.037	−0.240
VLTH (mm)	−0.016	0.094	−**0.487**	0.132	0.132	0.235	−0.070	−0.022	−0.043	0.131	0.008	−0.019	−0.182	−0.157	0.039	0.175	0.064	0.050
FLTH (mm)	−0.085	−0.217	0.136	−**0.473**	−0.178	0.165	0.097	0.466	0.946	−0.875	0.030	0.040	0.364	−0.075	−0.071	0.316	−0.067	−0.377^*^
VLT (°C)	−0.093	−0.263	0.210	−0.274	−**0.306**	0.094	0.105	0.421	1.334	−1.313	0.022	0.043	0.258	0.075	−0.056	0.035	−0.104	−0.221
FLT (°C)	−0.069	−0.047	−0.097	−0.066	−0.025	**1.177**	−0.057	0.044	−0.860	0.175	−0.036	−0.013	−0.167	−0.171	0.028	0.257	−0.047	−0.079
FT (days)	−0.012	−0.217	0.136	−0.184	−0.129	−0.271	**0.249**	0.277	1.161	−1.007	−0.025	0.038	0.091	0.171	−0.041	−0.222	−0.020	−0.016
PH (cm)	0.243	0.310	−0.010	0.199	0.116	−0.047	−0.062	−**1.109**	−0.258	0.438	0.085	−0.024	−0.061	0.142	0.013	−0.292	0.151	0.309
TN	0.117	−0.082	−0.005	0.104	0.095	0.235	−0.067	−0.067	−**4.302**	4.246	0.033	−0.029	−0.349	−0.224	0.060	0.316	0.062	0.247
PN	0.129	−0.070	−0.015	0.095	0.092	0.047	−0.057	−0.111	−4.173	**4.377**	0.052	−0.026	−0.364	−0.238	0.058	0.339	0.081	0.267
PL (cm)	0.061	−0.018	−0.015	−0.052	−0.025	−0.153	−0.022	−0.344	−0.516	0.832	**0.275**	0.007	0.045	0.179	−0.030	−0.222	0.091	0.199
PB	−0.040	−0.158	0.107	−0.213	−0.150	−0.177	0.107	0.299	1.420	−1.269	0.022	**0.088**	0.667	0.224	−0.110	−0.070	−0.037	0.111
SB	−0.012	−0.170	0.058	−0.114	−0.052	−0.129	0.015	0.044	0.989	−1.050	0.008	0.039	**1.511**	0.335	−0.193	0.023	0.030	0.115
FGN	0.000	−0.047	0.102	0.047	−0.031	−0.271	0.057	−0.211	1.291	−1.401	0.066	0.026	0.682	**0.745**	−0.135	−0.935	0.039	0.377^*^
%SF	−0.032	−0.211	0.088	−0.156	−0.080	−0.153	0.047	0.067	1.204	−1.182	0.039	0.045	1.364	0.470	−**0.215**	−0.140	0.015	0.039
TGN	0.032	0.111	0.073	0.128	0.009	−0.259	0.047	−0.277	1.161	−1.269	0.052	0.005	−0.030	0.596	−0.026	−**1.169**	0.037	0.372^*^
BM (g)	0.206	0.088	−0.127	0.128	0.129	−0.224	−0.020	−0.676	−1.075	1.444	0.102	−0.013	0.182	0.119	−0.013	−0.175	**0.247**	0.482^**^
**DRY SEASON 2013 NORMAL SOWN CONDITIONS**																		
VLS	**0.302**	−0.163	0.001	−0.043	−0.004	0.043	0.003	0.023	0.028	−0.094	0.134	−0.160	−0.040	0.036	0.602	0.097	−0.214	0.116
FLS	0.191	−**0.259**	0.008	−0.021	−0.003	0.108	−0.001	0.033	0.003	−0.015	0.113	−0.127	−0.019	0.021	0.332	0.054	−0.285	−0.058
VLTH (mm)	0.006	−0.039	**0.055**	0.159	0.004	0.041	0.001	0.001	−0.011	0.037	0.071	−0.028	0.007	0.003	0.037	0.016	−0.131	0.192
FLTH (mm)	0.030	−0.013	−0.020	−**0.429**	−0.003	−0.003	−0.022	−0.009	0.012	−0.047	0.054	−0.271	−0.027	0.032	0.504	0.081	0.077	−0.356^*^
VLT (°C)	−0.057	0.047	0.010	0.056	**0.019**	−0.035	0.003	0.021	0.003	−0.007	−0.196	0.066	0.020	−0.003	−0.074	−0.021	−0.036	−0.093
FLT (°C)	−0.048	0.103	−0.008	−0.004	0.003	−**0.271**	0.004	−0.027	−0.012	0.035	−0.017	−0.006	−0.009	−0.001	−0.061	−0.107	0.285	0.059
FT (days)	−0.006	−0.003	−0.001	−0.069	0.000	0.008	−**0.140**	−0.022	−0.002	0.007	0.075	−0.221	−0.032	0.027	0.332	−0.113	0.202	0.095
PH (cm)	−0.082	0.101	−0.001	−0.043	−0.005	−0.084	−0.036	−**0.086**	0.002	−0.005	0.088	−0.138	−0.011	0.006	0.074	0.027	0.374	0.220
TN	−0.109	0.010	0.008	0.064	−0.001	−0.041	−0.004	0.003	−**0.078**	0.246	0.038	0.006	0.021	−0.018	−0.369	−0.172	0.036	0.081
PN	−0.115	0.016	0.008	0.081	−0.001	−0.038	−0.004	0.002	−0.077	**0.248**	0.025	0.028	0.023	−0.023	−0.442	−0.166	0.036	0.064
PL (cm)	0.097	−0.070	0.009	−0.056	−0.009	0.011	−0.025	−0.018	−0.007	0.015	**0.418**	−0.315	−0.053	0.044	0.688	0.113	0.042	0.494^**^
PB	0.088	−0.059	0.003	−0.210	−0.002	−0.003	−0.056	−0.021	0.001	−0.012	0.238	−**0.553**	−0.056	0.063	0.946	0.070	0.077	0.103
SB	0.145	−0.059	−0.004	−0.137	−0.005	−0.030	−0.054	−0.011	0.020	−0.070	0.267	−0.371	−**0.083**	0.068	1.045	0.075	0.143	0.421
FGN	0.130	−0.065	0.002	−0.163	−0.001	0.003	−0.045	−0.006	0.017	−0.070	0.221	−0.415	−0.067	**0.084**	1.204	−0.048	0.012	0.415^**^
%SF	0.148	−0.070	0.002	−0.176	−0.001	0.014	−0.038	−0.005	0.023	−0.089	0.234	−0.426	−0.070	0.082	**1.229**	0.064	0.006	0.378^*^
TGN	−0.054	0.026	−0.002	0.064	0.001	−0.054	−0.029	0.004	−0.025	0.077	−0.088	0.072	0.012	0.008	−0.147	−**0.537**	−0.012	0.128
BM (g)	−0.109	0.124	−0.012	−0.056	−0.001	−0.130	−0.048	−0.054	−0.005	0.015	0.029	−0.072	−0.020	0.002	0.012	0.011	**0.594**	0.297
**DRY SEASON 2013 LATE SOWN CONDITIONS (HEAT STRESS)**																		
VLS	**0.125**	0.014	−0.026	0.011	−0.003	−0.002	0.001	0.027	−0.288	0.356	−0.092	0.016	0.024	−0.012	−0.014	0.104	−0.004	0.253
FLS	0.045	**0.040**	0.030	0.033	−0.003	0.013	−0.005	0.025	−0.039	0.073	−0.074	−0.005	−0.002	0.018	0.010	−0.005	−0.005	0.136
VLTH (mm)	−0.021	0.008	**0.156**	−0.002	−0.010	0.001	−0.022	0.022	0.118	−0.106	0.069	−0.033	−0.037	0.074	0.043	−0.233	0.002	−0.010
FLTH (mm)	0.006	0.006	−0.002	**0.219**	0.004	−0.005	−0.001	0.005	0.068	−0.073	−0.051	−0.003	−0.003	0.006	0.003	−0.010	0.003	0.168
VLT (°C)	−0.012	−0.004	−0.058	0.033	**0.026**	−0.008	0.007	−0.042	0.028	−0.046	0.051	0.005	0.005	−0.089	−0.011	−0.089	−0.002	−0.211
FLT (°C)	0.004	−0.007	−0.002	0.013	0.003	−**0.075**	0.014	0.031	0.000	0.020	−0.023	0.007	0.003	−0.031	0.006	−0.070	−0.001	−0.117
FT (days)	−0.001	0.004	0.065	0.004	−0.003	0.020	−**0.052**	−0.009	−0.130	0.139	0.055	−0.041	−0.018	0.083	0.028	−0.159	0.004	−0.029
PH (cm)	−0.030	−0.009	−0.030	−0.009	0.010	0.020	−0.004	−**0.114**	0.102	−0.092	0.267	−0.035	−0.020	0.080	0.013	−0.040	0.001	0.114
TN	0.064	0.003	−0.033	−0.026	−0.001	0.000	−0.012	0.020	−**0.564**	0.646	−0.064	0.013	0.023	0.000	−0.017	0.114	0.004	0.187
PN	0.067	0.004	−0.025	−0.024	−0.002	−0.002	−0.011	0.016	−0.553	**0.660**	−0.041	0.009	0.021	0.021	−0.014	0.119	0.004	0.272
PL (cm)	−0.025	−0.006	0.023	−0.024	0.003	0.004	−0.006	−0.066	0.079	−0.059	**0.461**	−0.053	−0.030	0.058	0.022	−0.164	0.000	0.200
PB	−0.026	0.003	0.070	0.009	−0.002	0.007	−0.029	−0.053	0.096	−0.079	0.332	−**0.074**	−0.041	0.163	0.049	−0.199	0.005	0.204
SB	−0.050	0.001	0.098	0.011	−0.002	0.004	−0.016	−0.039	0.220	−0.231	0.235	−0.052	−**0.059**	0.160	0.060	−0.258	0.001	0.044
FGN	−0.005	0.002	0.037	0.004	−0.008	0.008	−0.014	−0.030	0.000	0.046	0.088	−0.039	−0.031	**0.307**	0.047	0.119	0.009	0.537^***^
%SF	−0.025	0.006	0.097	0.011	−0.004	−0.007	−0.021	−0.022	0.141	−0.132	0.147	−0.052	−0.051	0.209	**0.069**	−0.253	0.004	0.068
TGN	0.026	0.000	−0.073	−0.004	−0.005	0.011	0.017	0.009	−0.130	0.158	−0.152	0.030	0.031	0.074	−0.035	**0.497**	0.003	0.514^**^
BM (g)	−0.027	−0.009	0.019	0.031	−0.003	0.004	−0.011	−0.007	−0.118	0.125	0.009	−0.019	−0.004	0.135	0.013	0.074	**0.020**	0.238

VLS, Vegetative leaf spad value; FLS, Flag leaf spad value; VLTH, Vegetative leaf thickness; FLTH, Flag leaf thickness; VLT, Vegetative leaf temperature; FLT, Flag leaf temperature; FT, Time for 50% flowering; PH, Plant height; TN, Tiller number per plant; PN, Panicle number per plant; PL, Panicle length; PB, Primary branches per panicle; SB, Secondary branches per panicle; FGN, Filled grain number per panicle; %SF, Percent spikelet fertility; TGN, Total grain number; BM, Biomass per plant; YPP, grain yield per plant. The values in bold are direct contribution of traits to YPP.

*** Significant at 0.001 level of probability;

** Significant at 0.01 level of probability;

* *Significant at 0.05 level of probability*.

### Single marker analysis

#### Marker trait associations in control and heat stress conditions during wet season 2012 and dry season 2013

**Figure 2:**
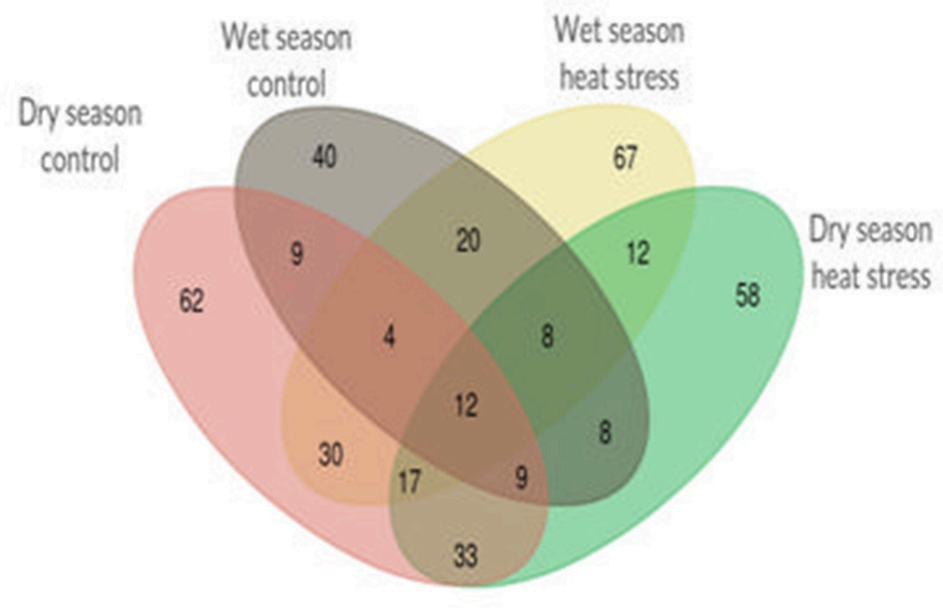
Venn diagram showing total 613 unique and shared significant marker-trait associations among normal and heat stress conditions during wet season 2012 and dry season 2013.

Single marker analysis was performed with 49 selected SSR markers and 18 agronomic and yield traits to find out significant (*p* < 0.05) marker-trait associations. In all, 613 significant marker trait associations were obtained, out of which 109 and 172 were from control and heat stress in wet season and 174 and 158 were from control and heat stress in dry season respectively. There were only 12 marker-trait associations, common in all four treatments (Figure 2). The list of significant marker-trait associations in each treatment and in each season is given in Supplementary Table 4. RM430 showed maximum number of associations (27) followed by RM440 and RM405 (26 each) and RM210 (25). The markers showing only one association were RM108 (with SB in WN), RM148 (with PB in WH) and RM349 (with VLS in DH). RM128 and RM406 showed two associations each. RM128 was associated with FLS and VLTH under WH and DH respectively and RM406 was associated with FLT and PH only under WH. Irrespective of treatments, YPP showed more number of associations (51) next only to PH (71) whereas FGN (12) and FLT (13) showed least associations. Out of 51 significant marker associations with YPP, four markers, RM106, RM225, RM440, and RM518 were associated with YPP in at least three treatments out of four. However, none were associated with YPP only under heat stress in both seasons. Likewise, there were 16 significant marker associations with %SF, out of which 11 associations were shared with TN, and TGN but only seven with YPP. All these seven common associations with YPP (two in control and five in heat stress) were observed only during dry season. It is noteworthy that there were no common marker associations with %SF and YPP in wet season.

### Marker-trait associations only under heat stress

The list of 11 common marker-trait associations found only under heat stress and in both wet and dry seasons involving nine markers and six traits is shown in Table 3. RM 243 was associated with both FLS and VLT whereas RM517 was associated only with FLS. RM518 and RM525 were also associated with VLT. RM225 was associated with FLTH and RM185 and RM282 were associated with plant height. Both panicle number and tiller number were associated with two common markers, RM489 and RM570. In addition to these common associations, there were 67 unique significant marker-trait associations only in WH, out of which more associations were with FLS (15) and VLT (11). Similarly, there were 58 unique significant marker-trait associations in DH, out of which more associations were with VLTH (13). In WH, only four unique associations (with markers RM 183, 332, 401, and 3735) were observed with YPP whereas there was only one unique association with YPP (with RM210) in DH.

**Table 3:** Marker trait associations observed only under heat stress conditions in both seasons (under polycover house during wet season 2012 and late sown conditions during dry season 2013).

**Trait**	**Marker**	**Chromosome**	**Polycover house (*F* value)**	**Late sown (*F* value)**
FLS	RM243	1	5.62^*^	5.15^*^
	RM517	3	4.94^*^	5.18^*^
FLTH	RM225	6	10.97^**^	6.60^*^
VLT	RM243	1	4.57^*^	6.93^*^
	RM518	4	4.43^*^	4.74^*^
	RM525	2	18.16^***^	7.13^*^
PH	RM195	8	4.58^*^	5.66^*^
	RM282	3	8.17^**^	6.91^*^
TN	RM489	3	10.55^**^	5.29^*^
	RM570	3	10.55^**^	5.29^*^
PN	RM489	3	8.61^**^	6.22^*^
	RM570	3	8.61^**^	6.22^*^

*** Significant at 0.001 level of probability;

** Significant at 0.01 level of probability;

* *Significant at 0.05 level of probability. FLS, Flag leaf spad value; FLTH, Flag leaf thickness; VLT, Vegetative leaf temperature; PH, Plant height; TN, Tiller number per plant; PN, Panicle number per plant*.

### Candidate genes

In all, 45 candidate genes which are known to respond to various stress conditions were identified within the region, from 1 Mb upstream to 1 Mb downstream of the nine markers significantly associated with six traits (flag leaf spad, flag leaf thickness, vegetative leaf temperature, plant height, panicle number and tiller number) under heat stress conditions in both wet and dry seasons (Supplementary Table 5). Out of these 45, nine are stress response genes, six are defense responsive genes, five are heat shock binding protein genes and four are related to oxidative stress response.

### Correlation of yield per plant (YPP) with other traits

Table 4 gives the list of traits correlated significantly with YPP based on Pearson correlation and list of traits sharing marker associations with YPP based on SMA. During wet season, YPP was correlated positively and significantly with BM, SB, and PL in control but it was correlated with BM, FGN and %SF in heat stress. SMA results showed four markers (RM88, RM106, RM274, and RM3586) associated with YPP in WN, out of which two were shared by BM (RM106 and RM274) and nine other traits shared only one common marker (FLTH, FLT with RM88, PB, PL, FLS with RM106, FT, PH, TN, PN with RM274). In WH, out of 17 markers associated with YPP, both BM and PH showed 14 common markers each and FLTH and FLS had 13 and 10 markers common, respectively. BM was strongly associated with YPP in wet season in both control and heat stress based on both correlation and SMA analysis. During dry season, YPP was correlated significantly with PL, FGN, and TGN in control and with FGN and %SF in heat stress. According to SMA, in DN, out of 21 YPP associated markers, FLTH and PH showed 16 and 9 common markers, respectively. TGN and BM also showed seven and six common markers with YPP. In DH, out of nine markers associated with YPP, seven, six and five markers were common with VLTH, TGN and %SF respectively. These results indicate that based on both correlation and SMA analysis, FGN was consistently associated with YPP under both treatments in dry season whereas %SF was associated with YPP only under heat stress in dry season. There was a significant negative correlation between YPP and FLTH in WH and DN conditions.

**Table 4:** Correlation between grain yield and other agronomic and yield traits based on Pearson correlation and single marker analysis (SMA).

**Season**	**Treatment**	**Pearson correlation**				**Number of markers associated with YPP**	**SMA**			
Wet season 2012	Normal conditions	BM (0.453^**^)	SB (0.397^*^)	PL (0.343^*^)		4	BM (2)			
	Polycover house conditions (heat stress)	BM (0.482^**^)	FGN (0.377^*^)	% SF (0.372^*^)	FLTH (-0.377^*^)	17	BM (14)	PH (14)	FLTH (13)	FLS (10)
Dry season 2013	Normal sown conditions	PL (0.494^**^)	FGN (0.415^**^)	TGN (0.378^*^)	FLTH (-0.356^*^)	21	FLTH (16)	PH (9)	TGN (7)	BM (6)
	Late sown conditions (heat stress)	FGN (0.537^***^)	%SF (0.514^**^)			9	VLTH (7)	UFGN (7)	TGN (6)	%SF (5)

The value in parenthesis are r2 value for Pearson correlation and no of markers associated in SMA.

*** Significant at 0.001 level of probability;

** Significant at 0.01 level of probability;

* *Significant at 0.05 level of probability. FLS, Flag leaf spad value; VLTH, Vegetative leaf thickness; FLTH, Flag leaf thickness; PH, Plant height; PL, Panicle length; SB, Secondary branches per panicle; FGN, Filled grain number per panicle; UFGN, Unfilled grain number per panicle; %SF, Percent spikelet fertility; TGN, Total grain number; BM, Biomass per plant; YPP, grain yield per plant*.

## Discussion

Changes in phenology in response to heat stress can reflect the interactions between stress environment and plants. In the present study, in heat stress conditions, tiller number, filled grain number, % spikelet fertility and grain YPP decreased whereas plant height increased significantly in both seasons. The effects of heat stress were not consistent in the two seasons for other traits such as flowering time, leaf spad, leaf temperature, biomass. In case of VLS, VLTH, and FLT the trait mean values were reduced significantly under WH but increased significantly under DH. The reason for these contrasting results in the two seasons might partly be due to more humidity in polycover house. Also during DH, plants were exposed to heat gradually from the beginning itself and had time to acclimatize slowly to heat unlike in polycover house where plants were covered at only flowering stage in wet season to give heat stress. Correspondingly, the percent reduction in trait values in DH was lesser than in WH when compared with their respective control conditions.

The present study showed that the mean %SF, BM, and YPP of all genotypes deceased under heat stress conditions. These results are in concurrence with earlier reports. High temperature at heading stage significantly reduced anther dehiscence and pollen fertility rate, leading to reduction in the number of pollen on stigma and subsequent reduction in spikelet fertility and yield in rice (Ahmad et al., 2010). High night temperature (32°C) led to increase in spikelet sterility (by 61% compared to control) in rice which resulted from decreased pollen germination (36%) (Mohammed and Tarpley, 2009). Grain yield decreased by 2–6% with increase in temperature by 1°C (Wu et al., 2016). High temperature reduces plant growth by affecting the shoot net assimilation rates and thus the total dry weight of the plant (Wahid et al., 2007). However, Hatfield and Prueger (2015) reported that even though grain yield was reduced due to high temperature but leaf area and vegetative biomass were not significantly affected. Oh-e et al. (2007) and Poli et al. (2013) reported that plant height was more in high temperature than in ambient temperature condition. The current study also showed increase in plant height with increase in temperature. Increase in plant height increases transpiration cooling effect and helps to avoid high temperature in mungbean and wheat (Kumar et al., 2011; Hasanuzzaman et al., 2013).

The performance of each genotype for 18 traits (Supplementary Table 3) indicates that different genotypes have different mechanisms to cope with heat stress as all top 10 high grain yielding genotypes did not show similar trait values such as biomass, % spikelet fertility, spad values, leaf temperature and leaf thickness under heat stress conditions. Tiller number and FGN were the common traits that contributed to more yield in both WH and DH treatments. Even in the same season (for example in WH), few high yielding genotypes (eg., K-16-3 and KMR3) maintained low VLT and high VLS and BM to achieve high YPP but other genotypes (e.g., K-103 and K-198) maintained low VLTH and BM and high %SF to achieve high YPP.

Heat tolerant rice genotypes that had relatively high grain yield under high temperature stress maintained lower leaf temperature and higher spad values than heat sensitive genotypes. Heat tolerant genotypes often have the ability to reduce leaf temperature leading to reduced transpiration rate and thus retain normal physiological functions of the leaves under heat stress. Leaf spad values increased under high temperature, and led to delay in grain filling phase (Cao et al., 2008; Jumiatun et al., 2016). Jumiatun et al. (2016) also reported that different rice genotypes with better grain yield under heat stress, show different responses as adaptation to high temperature. For example, IR 64 showed the lowest leaf temperature, but Menthik Wangi and Jatiluhur showed well-exerted panicles perhaps to lower panicle temperature but the panicle temperature was not mentioned in this report. Plants which can produce many leaves around panicles can withstand high temperature because anther dehiscence is not adversely affected and benefits panicles due to transpiration cooling effect of leaves (Shah et al., 2011).

High temperature stress reduces yield indirectly through affecting various yield components. In the present study, both Pearson correlation and path coefficient analysis were performed to measure direct and indirect effect of component characters on YPP. In both seasons, in control, PL, TGN, and BM showed significant positive correlation with YPP whereas in heat stress, only %SF and FGN showed significant positive correlation. Yield per plant (YPP) and FLTH were negatively correlated but not consistent across treatments. In WN conditions, FTH, PH, TN, SB, TGN showed significant direct effect on grain yield while FGN showed significant direct negative effect. However, in WH, FGN showed significant positive direct effect on yield as did FLT, PN, and SB on yield. Based on both correlation and path coefficient analysis results, the major traits that could be exploited in breeding programmes for more grain yield are PL, BM, SB, and FGN in control and %SF, FGN, TGN, SB, and TN in heat stress. Mishra et al. (2015) and Reddy et al. (2013) also reported that based on correlation and path-coefficient analysis on grain and its related components in rice genotypes, biological yield per hill, harvest index and number of spikelets per panicle were major contributing characters to rice grain yield and could be depended on for selection of genotypes to increase genetic yield potential of rice. Greater yield can be attained by increasing total crop biomass, as there is a possibility to increase the allocation of that biomass toward grain production (Evans and Fischer, 1999; Peng et al., 2004). Total crop biomass production mainly depends on the balance between photosynthetic gains and respiratory losses, which in turn are greatly influenced by temperature (Yoshida, 1981). Number of panicles and harvest index are good indictors of indirect selection for grain yield due to their high direct effects and significant correlation with grain YPP in rice under warm conditions in Khuzestan (Moosavi et al., 2015).

Previously we reported association analysis with four traits only viz., % spikelet fertility, grain YPP and their heat susceptibility index (Prasanth et al., 2016). More agronomic traits are considered in this paper. In all, 613 marker-trait associations were observed using 48 selected SSR markers and 19 traits based on SMA. Out of 613, more number of unique associations (67) were observed in WH condition whereas more number (33) of shared associations were between DN and DH conditions. The common marker-trait associations observed under high temperature conditions in both wet and dry season are given importance as they were associated with traits only under heat stress conditions. RM225 on chromosome 6 was associated with flag leaf thickness. Xiao et al. (2011) reported two QTLs *qPF4* and *qPF6* between RM5687 and RM471 on chromosome 4 and between RM190 and RM225 on chromosome 6 affecting pollen fertility in recombinant inbred lines derived from a cross between a heat tolerant rice cultivar 996 and a sensitive cultivar 4628. Thus, RM225 is a common marker. In our study, both panicle number and tiller number were associated with two markers RM489 and RM570 on chromosome 3. Plant height was associated with RM195 on chromosome 8 and RM282 on chromosome 3 while flag leaf spad was associated with RM243 on chromosome 1 and RM517 on chromosome 3. These markers were reported to be associated with spikelet fertility in previous studies on heat stress (Cao et al., 2003; Zhang et al., 2008). RM570 is also reported to be linked with a gene Os03 g62910 involved in heading date postponement and development of wider and thicker leaves in rice (Yu et al., 2013). Thus, leaf thicknes and width appear important traits in the context of heat tolerance. RM282 was reported to be linked with spikelet number per panicle under moderate and extreme drought stress conditions in field (Mei et al., 2004). RM525 on chromosome 2 was associated with VLT in the present study. Liu et al., 2015 also reported that RM525 was associated with grain filling rate at two stages, 7 and 28 days after flowering during their studies of time-course association mapping on grain filling rate in 96 rice genotypes. Grain filling rate is influenced by temperature and we show that the same region of RM525 is associated with leaf temperature.

In the present study, 45 candidate genes were identified close to the nine markers significantly associated with six traits (flag leaf spad, flag leaf thickness, vegetative leaf temperature, plant height, panicle number and tiller number) under heat stress conditions in both wet and dry seasons. These genes include HSPs, *OsTIL-2*, Glyoxalase II, OsSPX1, TPR-1, and NAM. *Hsp70* and *DnaJ* were reported to be highly up-regulated under heat stress in panicles of heat tolerant rice cultivar 996 (Zhang et al., 2012). The promoter region of *OsTIL-2* on rice chromosome 8 has several heat shock elements (Charron et al., 2005). Chi et al., 2009 reported that TILs (Temperature induced lipocalin) are required for basal and acquired thermo-tolerance in Arabidopsis and act against lipid peroxidation induced by high temperature. Glyoxalase II is required for abiotic stress response including heat stress in Arabidopsis (Devanathan et al., 2014). OsSPX1 is a gene for semi male sterility whose down regulation causes sensitivity to cold and other oxidative stresses in rice (Wang et al., 2013).

These genes are reported to be putatively functional under heat stress conditions (http://rapdb.dna.affrc.go.jp/) and are candidate genes for expression studies and evaluation for use in marker assisted selection for heat tolerance.

## Conclusion

Heat tolerance is a complex phenomenon which may be species specific, tissue specific and even developmental stage specific. Thus, heat tolerance should not be regarded as a single trait. In the present study, correlation and path coefficient analysis showed that the major traits that contribute to heat tolerance in rice are TN, SB, TGN, FGN, and %SF. These traits can be considered important while screening rice genotypes for heat tolerance. This study also emphasized the importance of elite × wild introgression lines in the development of heat stress tolerant rice varieties. Three ILs (K-377-24, K-16-3, and S-148) were identified as heat tolerant and another three lines (K-363-12, S-75, and Vandana) were identified as heat susceptible based on YPP in both WH and DH conditions. These selected lines could be exploited in further breeding and genomic studies on rice heat stress. Based on SMA, 12 significant marker-trait associations which were common under heat stress conditions in both wet and dry seasons are identified as high priority regions for basic and applied studies on heat tolerance. Thus, we identified markers and genes which may be useful for marker assisted selection and also in functional genomics for discovery of genes important in increasing heat tolerance of rice crop.

## Author contributions

The present study was done under the guidance of SN. SN and VVP designed the experiment. VVP, MSB, and VGNTV carried out experiment and collected the data. RKB did data analysis and wrote manuscript. SN, SRV, and SKM corrected the manuscript.

### Conflict of interest statement

The authors declare that the research was conducted in the absence of any commercial or financial relationships that could be construed as a potential conflict of interest.
